# Regular breakfast consumption associated with high intelligence quotient: Myth or Reality?

**DOI:** 10.12669/pjms.315.7728

**Published:** 2015

**Authors:** Aliya Hisam, Mahmood Ur Rahman, Syed Fawad Mashhadi, Azfar Bilal, Tayyeba Anam

**Affiliations:** 1 Dr. Aliya Hisam, MBBS, MPH. Assistant Professor, National University of Science & Technology (NUST), Community Medicine Department, Army Medical College (AMC), Abid Majeed Road, Rawalpindi, Pakistan; 2 Mahmood Ur Rahman, MBBS, DPH, MPH, MSc, FCPS. Professor and Head of Community Medicine Department, Community Medicine Department, Army Medical College (AMC), Abid Majeed Road, Rawalpindi, Pakistan; 3 Syed Fawad Mashhadi, MBBS, MPH, MPhil, MCPS. Assistant Professor, Community Medicine Department, Army Medical College (AMC), Abid Majeed Road, Rawalpindi, Pakistan; 4 Azfar Bilal, House Officers, Military Hospital, Rawalpindi, Pakistan; 5 Tayyeba Anam, House Officers, Military Hospital, Rawalpindi, Pakistan

**Keywords:** Breakfast, Intelligence, Students

## Abstract

**Objective:**

To find the frequency of regular breakfast consumption among Pakistani teenagers while the other objective was to find out the association between breakfast consumers (BC) and intelligence quotient (IQ).

**Methods:**

This comparative cross sectional study was conducted on 102 students of a Public School Rawalpindi from August 2013 to January 2014. Participants were categorised into two groups i.e. regular breakfast consumers (RBC) and irregular breakfast consumers (IBC) according to their breakfast habits. A standardized questionnaire of Wechsler Intelligence Scale for Childrenwas used for IQ assessment. Data was then entered and analysed in SPSS version 20.

**Result:**

Out of the 102 individuals with mean age 17.17 ± 0.631, 58(56.9%) were females and 44 (43.1%) were males. There were 63 (61.8%) RBC while 39 (38.2%) were IBC. Among RBC there were 7 (6.9%) in challenged, 5 (4.9%) were below average, 33 (32.4%) in average group, 14(13.7%) in above average and 4 (3.9%) in gifted group. While among IBC, there was 1 (1%) among the severely challenged, 3 (2.9%) in challenged, 8 (7.8%) in below average, 22 (21.6%) in average group, 4 (3.9%) in above average and 1 (1%) in gifted group. There was no significant association found between breakfast intake and IQ level among students (p=0.98).

**Conclusion:**

More than half of the students were having regular breakfast. There was no significant association found among breakfast consumers and IQ. However the IQ score was more among RBC as compared to IBC.

## INTRODUCTION

Diet is one of the dominant and preventable health problems. Adoption of healthyl food intake patterns is an essential foundation for chronic disease prevention and maintenance of a healthy life style.^1^ Breakfast refers to the 1^st^ meal which is taken in the morning and taken before the start of daily activities and is often regarded as the most important meal of the day. Breakfast means breaking-the-fast of the night.^2^

Childhood is a critical period in which dietary and lifestyle patterns are initiated. Childhood habits can have important immediate and long-term implications. Breakfast habits appear to be no exception^3^ but approximately one in four to five college students skip breakfast.^1^ Skipping breakfast or consuming an inadequate breakfast contributes to dietary inadequacies that are seldom compensated for at other meals.^3^ Breakfast contributes to the quality and quantity of a person’s daily dietary intake. Overnight and morning fast may influences the problem-solving performance of well-nourished children.^4^ In United States, there is increasing evidence that eating breakfast can yield many health benefits for growing children, ranging from improved overall dietary quality to enhanced classroom performance.^3^

Intelligence quotient is a number representing a person’s reasoning ability (measure dosing problem-solving tests) as compared to the statistical norm or average for their age, taken as 100.^5^ Intelligence is related to academic scores and class performance.^6^ IQ of school going children is affected by many factors including mothers diet, socioeconomic factors, parental education, eating behaviours etc.^7^ Childhood habits can have important immediate and long-term implications. Breakfast habits appear to be no exception. Irregular breakfast eating has also been associated with a number of unhealthy behaviours, such as smoking, frequent alcohol use, and infrequent exercise.^8^

There is a consensus regarding the universal significance of breakfast (BF) for health, wellbeing, and cognition.^9^ Individual cognition and a supportive home environment are associated with adolescent breakfast consumption.^10^ Regular breakfast consumption can have a multitude of positive health benefits, yet young people are more likely to skip breakfast than any other meal. Dietary behaviours established in childhood and adolescence track into adulthood and also the habit of breakfast skipping increases with age. Breakfast behaviours of children and adolescents are complex and influenced by multiple factors. It is important to highlight the potential importance of the family for the promotion of breakfast consumption among young people.^11^

Studies^4^,^7^,^8^,^13^ regarding breakfast intake and IQ or cognitive ability indicating positive association between regular intake and improved performance have been done in different part of the world. In our part of the world, there is lack of knowledge regarding the dietary habits of teenager as well as the effect of regular breakfast intake with intelligence quotient. As in our setup, the nutritional value of the food is expected to be compromised because of low food quality, low standards of food inspection; there is a need to find out the frequency of breakfast-skipping on a regular school day of young age group: to assess the association between regular breakfast intake and IQ and also to explore the reasons behind skipping breakfast. It is essential to identify the high risk teenage groups and make them aware regarding the ill effects of skipping breakfast.

## METHODS

A comparative cross sectional study was conducted at a public school in Rawalpindi from August 2013 till January 2014. Using WHO sample size calculator, the sample size was calculated to be approximately 102 with confidence level (CL) of 95%, anticipated population proportion (P) of 0.90 and absolute precision (d) of 0.065. Purposive sampling technique was used. Boys and girls of 12^th^ grade were inducted in the study. Students who were not willing or were having any acute or chronic disease were excluded from the study sample. Permission from the ethical committee was taken. Assent from the Principal and informed consent from the student was taken. After taking demographic and breakfast intake information, the students were divided into two groups: RBC (students having 5 or more than 5 breakfast per week) and IBC (students having 4 or less than 4 times breakfast per week). Then a standardized questionnaire of Wechsler Intelligence Scale^12^ for Children was (consisting of 20 questions) was given to all of them to fill in 20 minutes. The questionnaire responses were later entered on the internet website to grade their IQ score. Individual grading was then noted in their respective questionnaire. Grading IQ Score^12^ was as follows:

**Table-I:** Intelligence Quotient Score Chart.

0-39	Severely challenged
40-69	Challenged
70-84	Below average
85-114	Average
115-129	Above average
130-144	Gifted
>144	Genius

Data was entered and analysed in SPSS version 20. Descriptive statistics was used to calculate mean and standard deviation for quantitative variables like age and IQ grade. Frequency and percentage are calculated for qualitative variables like gender, breakfast consumers, reasons for skipping breakfast and IQ grading. Chi square test was applied to find out association of IQ grade and breakfast consumption.

## RESULTS

One hundred and two students were inducted in the study. Participants mean age was 17.17 ± 0.631. There were 58 (56.9%) females and 44 (43.1%) males.

When inquired regarding their breakfast frequency, 63 (61.8%) were having breakfast 5-6 times, 16 (15.7%) 4-5 times, 15 (14.7%) 2-3 times and 8 (7.8%) were having breakfast 0-1 times per week. Based upon operational definitions, there were 63 (61.8%) regular breakfast consumers (RBC) identified while 39 (38.2%) were irregular breakfast consumers (IBC). Out of RBC, 31(49.2%) were females while 32 (50.8%) were males. Among IBC, 27 (69.2%) were females and 12 (30.8%) were males. There was a significant association found between gender and breakfast consumption (p=0.047).

When questioned regarding the reasons for skipping breakfast (n=102), only 40 (39.2%) use to have breakfast daily. Among the rest of the 62 participants, the most common reason for skipping breakfast was not feeling hungry in the morning that is about 23 (22.5%). Second most common reason, 15 (14.7%) was getting late for school. About 11 (10.8%) replied that they did not wanted to have anything in the morning. Only 3 (2.9%) replied that they were too lazy to have anything in the morning while 10 (9.8%) had no specific reason for skipping breakfast. Details are shown in Table-II.

**Table-II:** Demographic characteristics of the study participants (n=102).

Demographic Variables	Regular Breakfast Consumer n=63 (61.8%)	Irregular Breakfast Consumer n=39 (38.2%)	Total Population n=102 (%)	p-value
Mean Age	17.17 ± 0.631			
*Gender*
• Male	32 (50.8%)	12 (30.8%)	44 (43.1%)	p=0.047*
• Female	31 (49.2%)	27(69.2%)	58 (56.9%)
*Reasons for skipping breakfast*
• Always eat	--	--	40(39.2%)	
• Not feeling hungry	4(3.9%)	19(18.6%)	23(22.5%)	
• Late for school	4(3.9%)	11(10.8%)	15(14.7%)	
• Did not wanted to eat	4(3.9%)	7(6.9%)	11(10.8%)	
• Too lazy	1(1%)	2(2%)	3(2.9%)	
• Others	10(9.8%)	0(0%)	10(9.8%)	--
*Intelligence Quotient*
• Severely Challenged	0 (0%)	1(1%)	1(1%)	
• Challenged	7(6.9%)	3(2.9%)	10(9.8%)	p=0.178
• Below Average	5(4.9%)	8(7.8%)	13(12.7%)	
• Above Average	33(32.4%)	22(21.6%)	55(53.9%)	
• Good Average	14(13.7%)	4(3.9%)	18(17.6%)	
• Gifted	4(3.9%)	1(1%)	5(4.9%)	

* Significant association.

Among the whole study population (n=102), 1 (1%) was severely challenged, 10 (9.8%) challenged, 13 (12.7%) below average, 55 (53.9%) average, 18 (17.6%) above average, 5 (4.9%) gifted and zero were genius.

**Fig.1:**
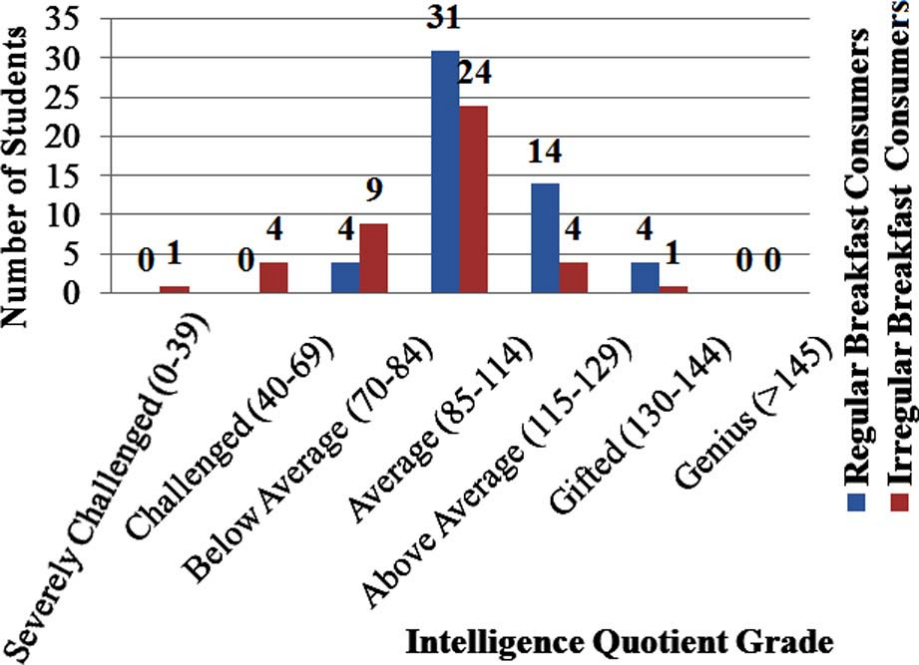
Association of breakfast with IQ grade among students (n=102) (p=0.178).

Among RBC, there were none among the severely challenged, 7 (6.9%) in challenged, 5 (4.9%) in below average group, 33 (32.4%) in average group, 14(13.7%) in above average, 4 (3.9%) in gifted group and none were genius. While among IBC, there was 1 (1%) among the severely challenged, 3 (2.9%) in challenged, 8 (7.8%) in below average group, 22 (21.6%) in average group, 4 (3.9%) in above average, 1 (1%) in gifted group and none were genius. There was no significant association found between breakfast intake and IQ level among students (p=0.178). Details are shown in Fig.1.

## DISCUSSION

Regular breakfast consumers were almost half among the Pakistani teenager students who were studied. Among breakfast skippers, the most common reason of skipping breakfast was not feeling hungry in the morning. Better IQ grade was observed among teenagers (both males and females) who regularly consumed breakfast as compared to those who irregularly consumed breakfast. A similar study conducted in university of Bristol, UK showed the same results as ours but it was conducted in people of older age group.^13^

College students have lack of knowledge or are less interested regarding their nutrition and also have undesirable nutritional habits. Academic performance is affected by not meeting the nutritional needs of a student.^14^ One of our study limitations was that we did not explored the knowledge regarding nutrition or importance of breakfast intake. If students have knowledge regarding the importance of their nutritional requirement when they start off their day, they might rectify the mistake of skipping breakfast.

A descriptive cross sectional study was conducted in Jintan, a Chinese city inducted a sample of 1269 children (697 boys and 572 girls) aged 6 years. They assessed the cognition with the Chinese version of the Wechsler Preschool and Primary Scale of Intelligence. Breakfast inquiries were done from the parents of the children. Children having almost always breakfast were having more good scales (verbal and performance) (p < 0.001) as compared to one who sometimes have it.^8^ As we recruited boys and girls of school and they were able to answer questionnaire related to breakfast habits so we inquired directly from the participants. This added strength to our study results and reduced information bias. We used a standardized questionnaire for evaluation of students intelligence which further reduced the bias. In contrast to a study in United Kingdom,^14^ our study results showed no significant association between breakfast consumption and IQ level (p=0.98). But among regular breakfast consumers, more students were having average, good, gifted IQ scoring as compared to the other group. A study in United States showed that a large percentage of children who skipped breakfast did not meet two-thirds of the recommended dietary allowances for vitamins and minerals.^3^ In Pakistan, are the teenagers meeting their daily dietary requirement? What is the quality of food in breakfast here? There is a need to investigate this aspect also.

About 76.7% of the primary school children were having regular breakfast intake at home while only 23.3% were not having breakfast, according to a study in Baghdad city. The average number of breakfast was 4.5 times per week.^15^ Seventy seven percent children also showed having high intelligence level as they scored more than 75th percentile. Our study showed results also show more trends towards breakfast consumption i.e. RBC being 63 (61.8%) and IBC being 39 (38.2%). But in contrast no significant association was found between BC and IQ. In UK, a population based study showed significant association between breakfast and well-being.^16^

About 1,782 5-year-old children were inducted in a study from the Danish National Birth Cohort (2003–2007). A short form of the Wechsler Preschool and Primary Scale of Intelligence was used as a data collection tool. Their result showed that parental education and maternal IQ are the major predictors of children IQ.^17^ Since parents primarily shape the home environment, interventions aimed at improving adolescent breakfast consumption should target the parent as well as the adolescent.^10^ Perhaps we have to target the parent simultaneously with teenagers to have a firm habit of regular breakfast. We inquired into the reasons of skipping breakfast out of which most common was not feeling hungry in the morning but still we need to explore more reasons behind skipping breakfast. Although no significant association was found but still the IQ grading was better in students having regular breakfast.

Is the quality of food consumed during breakfast responsible for not having significant association between breakfast consumption and IQ grade? We need our youth to be intelligent for which we need to dig deeper down for risk factors associated with irregular breakfast consumption. If we could find significant factors associated in further studies, we could target those factors and acquire primordial prevention strategies for this uncovered aspect of intelligence among youth.

### Limitations of the study

An important limitation of this study is that we cannot draw any causal inference between RBC and IQ. Though there was no significant association established between RBC and IQ but we should acknowledge that it is also possible that poor IQ could lead to poor breakfast consumption and vice versa.

## CONCLUSION

More than half of the students were having regular breakfast. There was no significant association found among breakfast consumers and IQ grade. However the IQ score was more good among regular breakfast consumers as compared to irregular breakfast consumers.

## RECOMMENDATIONS

There is a need that parents and care providers are encouraged that they should become a part of health promotion activities and try to encourage their offspring to adopt healthy lifestyles with inclusion of daily breakfast. Health education regarding importance of regular breakfast intake must be given by media, mass campaign through NGO’s and community-based programmes in schools and colleges. It can also be made a part of MCHC services where mothers are educated regarding the importance of healthy regular breakfast.
